# Operationalizing a patient engagement plan for health research: Sharing a codesigned planning template from a national clinical trial

**DOI:** 10.1111/hex.13417

**Published:** 2021-12-24

**Authors:** Holly Etchegary, Andrea Pike, Andrea M. Patey, Erin Gionet, Brian Johnston, Susan Goold, Vanessa Francis, Jeremy Grimshaw, Amanda Hall, De‐Implementing Wisely Research Group

**Affiliations:** ^1^ Clinical Epidemiology Program, Faculty of Medicine, Patient Engagement Lead, NL SUPPORT, CIHR‐SPOR, Craig L. Dobbin Centre for Genetics Memorial University St. John's Newfoundland and Labrador Canada; ^2^ Primary HealthCare Research Unit, Faculty of Medicine Memorial University St. John's Newfoundland and Labrador Canada; ^3^ Centre for Implementation Research Ottawa Hospital Research Institute Ottawa Ontario Canada; ^4^ Patient Engagement Coordinator (CWC‐iCT) Cumming School of Medicine University of Calgary Calgary Alberta Canada; ^5^ Patient Partnership Council (CWC‐iCT), Quality of Care NL/Choosing Wisely NL Memorial University St. John's Newfoundland and Labrador Canada; ^6^ Patient Partnership Council, (CWC‐iCT), Quality of Care NL Memorial University St. John's Newfoundland and Labrador Canada; ^7^ Patient Partnership Council, (CWC‐iCT) Memorial University St. John's Newfoundland and Labrador Canada; ^8^ Department of Medicine, Centre for Implementation Research, Ottawa Hospital Research Institute University of Ottawa Ottawa Ontario Canada

**Keywords:** levels of engagement, patient engagement, patient‐oriented research, planning tool

## Abstract

**Introduction:**

Engaging with patients about their lived experience of health and illness and their experience within the healthcare system can help inform the provision of care, health policies and health research. In the context of health research, however, operationalizing the levels of patient engagement is not straightforward. We suggest that a key challenge to the routine inclusion of patients as partners in health research is a lack of tangible guidance regarding how this can be accomplished.

**Methods:**

In this article, we provide guidance on how to codesign and operationalize a concrete patient engagement plan for any health research project.

**Results:**

We illustrate a seven‐step approach using the example of a national clinical trial in Canada and provide a patient engagement planning template for use in any health research project.

**Conclusion:**

Such concrete guidance should improve the design and reporting of patient engagement in health research.

**Patient or Public Contribution:**

The De‐Implementing Wisely Research group is informed by a national 9‐member patient partner council (PPC). The research team includes three lead patient partners who are coinvestigators on the grant that funds the program of research. Members of the council advise on all aspects of the study design and implementation. The ideas presented in this paper were informed by regular communication and planning with the PPC; specific contributions of lead patient partner authors are outlined as follows: Brian Johnston, Susan Goold and Vanessa Francis are patient partners with a wide breadth of experience in the healthcare system and health research projects. The guidance in this article draws on their lived and professional expertise. All patient partner authors contributed to the planning of the manuscript, participated in meetings to develop content and provided critical manuscript edits and comments on drafts.

## 1 INTRODUCTION

Few would argue that engaging with patients about their lived experience of health and illness and their experiences within the healthcare system can help inform the provision of care, health policies and health research. In the context of a health research project, however, what exactly does patient engagement mean? How can researchers *partner* with patients in the design and conduct of health research, rather than involve them solely as study participants? Despite the growing consensus on the value of patient engagement in health research, tangible examples of how to create a patient engagement plan (PEP) remain limited. In this article, we provide guidance on how to codesign and operationalize a detailed PEP for any health research project. We illustrate a seven‐step approach using the example of a national clinical trial in Canada and offer lessons learned in the process. We propose this guide as a resource for research funders, researchers, providers and patient partners with the goal of advancing a more rigorous approach to the development and implementation of patient engagement in health research.

### 1.1 A growing consensus: Patient engagement in health research

Multiple frameworks, best practices, checklists and questions to guide research teams now exist for patient engagement,^1^, ^2^, ^3^, ^4^, ^5^, ^6^, ^7^ driven in part by the call from leading funders and journals to partner with patients in the conduct of health research.^6^, ^8^, ^9^, ^10^ Evidence suggests that partnering with patients in the design and conduct of health research can improve both research quality and outcomes.^11^, ^12^, ^13^ For example, patient engagement helps ensure that research questions and study measures reflect patient‐identified priorities.^11^ Engagement has helped increase study recruitment and contributed to the design of informed consent documents and other study materials, as well as helped the translation of research findings into practice.^11^, ^12^, ^13^, ^14^ Engagement occurs across all stages of the research process from conceptualisation of study questions and protocols, to defining and choosing study measures and outcomes, to helping to collect or analyse study data, to knowledge translation and dissemination efforts.^5^, ^8^, ^11^, ^12^, ^14^

### 1.2 Levels of involvement

**Figure 1:**
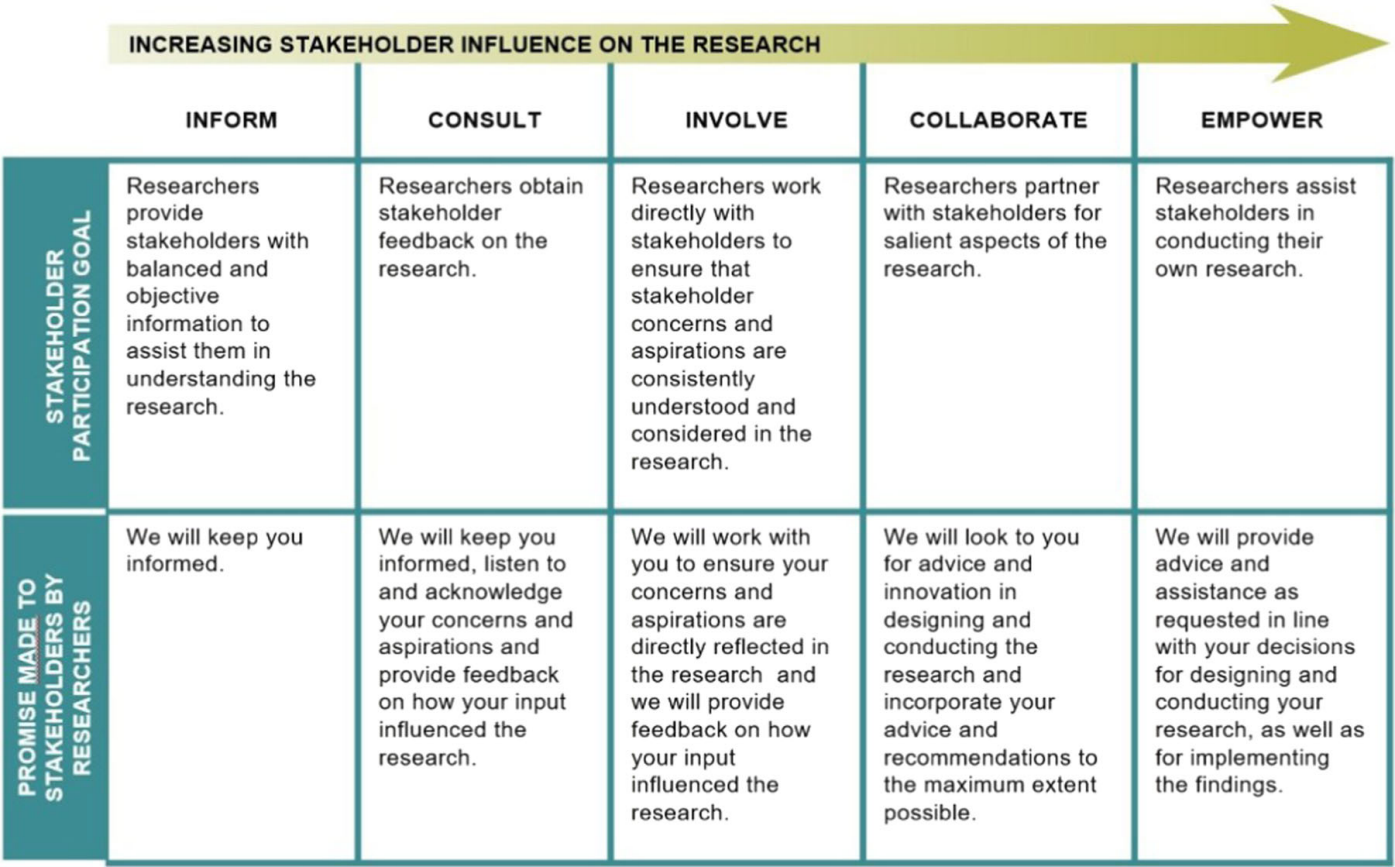
The research‐modified International Association for Public Participation (IAP2) spectrum. *Source*: Publicly available online by Gabriele Bammer, https://i2insights.org/2020/01/07/research-modified-iap2-spectrum/

Patient engagement is often described on a continuum from lower levels of ability to influence decisions to higher levels that provide decision‐making authority.^5^, ^15^ The International Association for Public Participation (IAP2) identifies five levels of engagement ranging from lesser to greater levels of engagement: inform, consult, involve, collaborate and empower.^15^ Conceptually, as one moves up the spectrum of engagement, greater levels of decision‐making power are available to patient partners. At the level of consult, for example, patients may be asked for their opinions about a project, but the use of their input is not guaranteed. At the level of collaboration, however, researchers commit to working with patient partners in each aspect of research decisions, including formulating decision options (e.g., what methods to use) and preferred solutions.^15^ It is becoming a well‐recognized and accepted standard for describing public involvement,^5^, ^6^ and has been research‐modified (Figure 1) to outline these levels and what they mean for engagement with stakeholders in research projects.^16^ It is a useful framework for thinking through how patients can be engaged at different levels for all health research project activities as we outline below.

While there are some exceptions,^17^ patient engagement in health research generally remains limited to preliminary activities or early stages of a project (e.g., identifying research priorities, helping with study recruitment), rather than later stages in the research lifecycle, such as data collection and analysis.^1^, ^5^, ^11^, ^13^, ^14^ This is largely related to barriers such as resource constraints (e.g., time, funding), but also limited availability or awareness of guiding frameworks and methods.^5^

### 1.3 Operationalizing patient engagement: Codesigning a PEP

Despite the growing body of research on patient engagement, there are few tangible examples in the literature demonstrating how the various levels of engagement can be operationalized in any given health research project. For example, what does it mean to ‘involve’ patients in choosing study measures or analysing data versus to ‘collaborate’ with them in such research activities? While useful resources for researchers^5^, ^6^ are available to help inform patient engagement planning, researchers and patient partners can benefit from more concrete guidance.

This paper describes a very tangible example of patient engagement in a large, national clinical trial in Canada. The goal is to share a practical template that can be used in any health research project, literally swapping out the information in the included example for others' relevant project information. This paper describes the process and lessons learned in codesigning the PEP. The template provided here should reduce the time and effort needed by research teams to create a PEP (thereby mitigating a substantial barrier to patient engagement).

## 2 METHODS

### 2.1 The research study that needed a PEP: Deimplementing low‐value care

The research project is funded through a Strategy for Patient‐Oriented Research (SPOR) Innovative Clinical Trial Multi‐Year Grant (GRANT #MYG‐158642). In Canada, the Canadian Institutes of Health Research (CIHR) oversees the SPOR, which is focused on ensuring that the right patient receives the right intervention at the right time and is committed to partnering with patients in the design and conduct of research.^10^ In the SPOR, patient engagement is defined as ‘meaningful and active collaboration in governance, priority setting, conducting research and knowledge translation’. Patients who actively contribute to research are defined as ‘patient partners’, including not just individuals with lived experience of a health condition, but also informal caregivers, including family and friends.^10^

Low‐value care, healthcare with little to no clinical benefit or that causes harm—is a challenge for healthcare systems globally, including Canada, resulting in direct harm to patients (due to adverse effects of treatments or secondary unwarranted tests) and threatening healthcare system sustainability.^18^, ^19^ Choosing Wisely Canada (CWC) is a national campaign engaging healthcare professionals and patients to identify low‐value care. CWC has developed a patient and public engagement framework and engaged in a wide range of activities to support patients and citizen involvement in CWC and increase awareness of the problem of low‐value care.^20^, ^21^

There remains considerable uncertainty about how best to deimplement low‐value care, suggesting the need for implementation research in this area. This project (see protocol in Grimshaw et al.^22^) uses the Choosing Wisely De‐implementation Framework and brings together CWC, Choosing Wisely provincial campaigns, patient and health system partners and researchers in three provinces (Alberta, Newfoundland and Ontario) to conduct six innovative cluster‐randomized trials of deimplementation strategies.

Two trials in each province will evaluate theoretically informed deimplementation strategies, targeting both healthcare professionals and patients to reduce two low‐value care practices (preoperative testing in low‐risk ambulatory surgery and imaging in uncomplicated low‐back pain). Here, the PEP for the study site in Newfoundland and Labrador (NL), Canada, is presented for 1 year of the low‐back pain trial.

### 2.2 The big picture of patient engagement in the deimplementing wisely project

Upon the advice of the study's three lead patient partners, a pan‐Canadian patient partner council was established to advise on all project activities. Two additional patient partners were recruited from each province, for a total of nine patient partners from three provinces in Canada. The council also comprises the project's patient engagement coordinator (E. G.), research lead (A. M. P.), and scientific patient engagement lead (H. E.) who act as nonvoting members. All council members collaboratively created an appreciation policy for the renumeration of patient partners, a Terms of Reference (ToR) for the group, specifically outlining the group's roles and expectations for the overall study, as well as some key patient engagement activities the council as a whole would engage in (e.g., providing early presentations to the rest of the team about patient engagement, contributing to a quarterly project newsletter, creating knowledge translation tools about patient engagement). The process for creating the ToR was iterative and included multiple discussions at initial council meetings and reviews of drafts by all council members with subsequent incorporation of their feedback by the patient engagement coordinator. Establishing the council, hiring the patient engagement coordinator and creating the initial ToR took a little over a year.

Once this initial relationship building and work had occurred, however, both researchers and patient partners acknowledged that uncertainty remained about how best to enact specific patient engagement activities for each trial, in each province. While the council's ToR was detailed and comprehensive, it remained challenging for the team to be concrete and explicit about specific roles and tasks.

### 2.3 Getting down to the specifics: Codesigning the PEP

Each provincial research team will work with their local patient partners to codesign a PEP for the two trials to be carried out in their province. The NL team took the lead on this task for the subproject on reducing imaging for nonspecific low‐back pain because of the patient engagement expertise of the local team. The AB and ON teams will replicate the process.

This approach was chosen after many months of struggling to articulate a complex patient engagement strategy for a multisite, multiyear research project. The team was challenged by varying research timelines across the three study sites. For example, the NL site had completed some data collection, while other sites were in the process of submitting ethics applications. The COVID‐19 pandemic further challenged timelines in the three provinces. Ultimately, the approach taken by the team was to develop a PEP for each trial area (preoperative testing in low‐risk ambulatory surgery and imaging in uncomplicated low‐back pain), for each province, and to do so for 1 year at a time. This approach allowed a clearer understanding of what project activities would realistically be undertaken in any given year and focused clearly on the activities related to one trial behaviour. Ultimately, this proved a much more feasible approach that not only outlined specific project activities for 1 year but also provided patient partners flexibility to be involved in the activities of interest to them, at a level with which they were comfortable. This also ensures that the PEP is reviewed at least once annually, the minimum we recommend (and more often in the event of unanticipated project changes).

## 3 RESULTS

Below, a seven‐step approach for engaging patients in research and codesigning a PEP is presented. A fillable PEP template is provided (Figure 2), while a fully worked example from 1 year of our trial is provided in Table 1. Note that in Table 1, we have used the position of the team member to indicate which team member is responsible for each activity and we indicate researcher time by initials. In cocreating PEPs within teams, however, we recommend identifying specific team members responsible for activities by name.

Figure 2Patient engagement planning tool template

**Figure:**
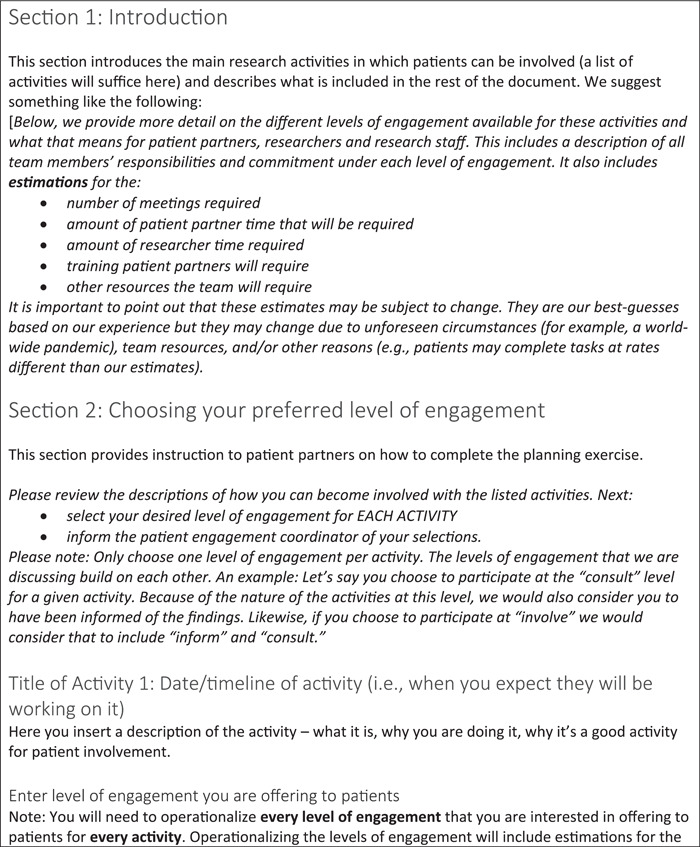

**Figure:**
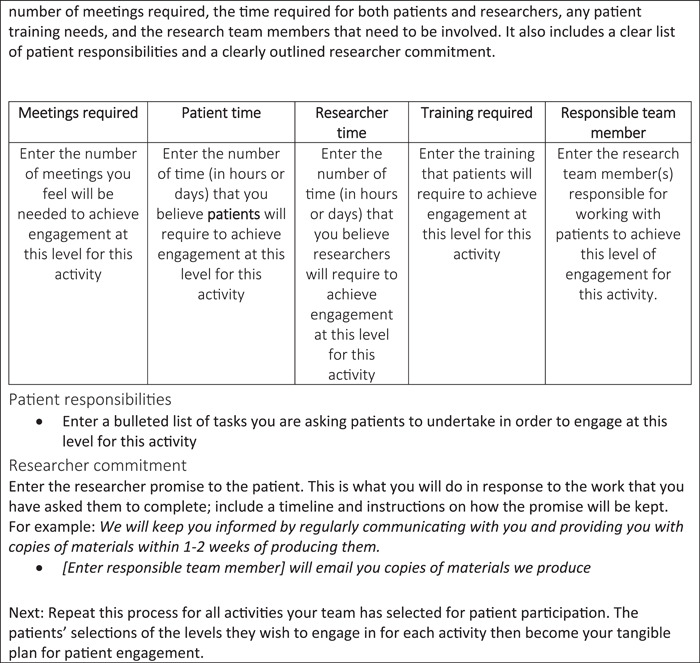

**Table 1:** Detailed patient engagement plan, innovative clinical trial low‐back pain study, Year 1

**Meetings required**	**Patient time**	**Researcher time**	**Training required**	**Responsible team member**
Barriers assessment—knowledge translation and dissemination				
September–October 2020				
This project activity includes helping to translate important messages about why we are trying to change practice and the key barriers to following low‐back pain guidelines that were found in our assessment. We want to translate the results from the barriers assessment into understandable focused messages that are relevant for the different knowledge users. The knowledge users for the study will be researchers, clinicians, patients and policymakers. Levels of patient engagement for this activity are outlined below.				
**Inform**				

**Meetings required**	**Patient time**	**Researcher time**	**Training required**	**Responsible team member**
0	Patient‐determined	–	None required	PE coordinator

**Meetings required**	**Patient time**	**Researcher time**	**Training required**	**Responsible team member**
Patient responsibilities				
Review the dissemination plan and related materials provided				
Researcher commitment				
We will keep you informed by regularly communicating with you and providing you with copies of materials within 1–2 weeks of producing them.				
The patient engagement coordinator will email you copies of materials we produce				
**Consult**				

**Meetings required**	**Patient time**	**Researcher time**	**Training required**	**Responsible team member**
0	30 min–1 h	A. P.—2 h	None required	Research Manager NL
		E. G.—1 h		PE coordinator

**Meetings required**	**Patient time**	**Researcher time**	**Training required**	**Responsible team member**
Patient responsibilities				
Review drafts of the key messages and dissemination materials crafted for each user group. Provide **written feedback** (emailed to Research Manager NL) on the accessibility/usability of the material from your perspective **within 1–2 weeks** of receiving these documents.				
Researcher commitment				
We will **consider** your feedback as we prepare the final versions of key messages and dissemination materials and let you know how your feedback influenced the process.				
Research Manager NL will email you draft copies of the materials we have produced (cc PE coordinator). PE coordinator will communicate to you how your input has influenced our work.				
**Involve**				

**Meetings required**	**Patient time**	**Researcher time**	**Training required**	**Responsible team member**
1	1.5–2 h	A. P.—3 h A. M. P.—1.5 h E. G.—1.5 h	None required	Research Manager NL Senior Research Manager, national site PE coordinator

**Meetings required**	**Patient time**	**Researcher time**	**Training required**	**Responsible team member**
Patient responsibilities				
Review drafts of the key messages and dissemination materials crafted for each user group. Provide **written feedback** on the accessibility/usability of the material from your perspective and help to generate a list of potential frequently asked questions **within 1–2 weeks** of receiving these documents.				
Researcher commitment				
We will work with you to ensure your feedback is incorporated and directly reflected in the finished product **to the maximum extent possible (while maintaining the integrity of the research)**.				
Research Manager NL will email you draft copies of the materials we have produced (cc PE coordinator). Selected members of the NL team will meet with you to review your feedback and discuss how we plan to incorporate it into the final product.				
**Collaborate**				

**Meetings required**	**Patient time**	**Researcher time**	**Training required**	**Responsible team member**
2–3	Up to 7 h (includes meeting hours and independent work)	A. P. A. M. P. 7—NL SUPPORT	Patient training (TDF)	Research Manager NL Senior Research Manager, National site PE Scientific Lead

**Meetings required**	**Patient time**	**Researcher time**	**Training required**	**Responsible team member**
Patient responsibilities				
Attend a lecture on the TDF. Partner with researchers on the team to translate the key messages and identify the most appropriate communication/dissemination methods. This includes: o participating in training on the theoretical domains framework, o attending meetings with selected team members to review the results, cocreate key messages based on those results and write the text for a 1‐page infographic report, o reviewing and commenting on subsequent drafts. Become a local champion of the key message by spreading the key message at different functions and through relevant personal networks if possible.				
Researcher commitment				
We will work with you to **cocreate** dissemination materials and select communication strategies, relying on your advice and innovation in this area. We will **incorporate your advice and recommendations to the maximum extent possible**.				
Research Managers and a local facilitator with meet with you to review the results of the barriers assessment and begin work on breaking the results down into key messages. Subsequent meetings will focus on developing the text for a 1‐page infographic report, selecting communication channels, and reviewing edits and feedback on various drafts of the report.				
**Trial—intervention development**				
September–December 2020				
Includes reviewing the problems identified in the barriers assessment, determining which problems were most important and able to be targeted and matching them with appropriate behaviour change techniques.				
**Inform**				

**Meetings required**	**Patient time**	**Researcher time**	**Training required**	**Responsible team member**
0	Patient‐determined	—	None required	PE coordinator

**Meetings required**	**Patient time**	**Researcher time**	**Training required**	**Responsible team members**
Patient responsibilities				
Review the behaviour change techniques that are selected to form the intervention.				
Researcher commitment				
We will keep you informed by telling you when the behaviour change techniques (BCTs) have been selected by the stakeholder committee.				
PE coordinator will email you correspondence **within 1–2 weeks** of making these decisions, indicating which behaviour change techniques have been selected to form our intervention and what barriers they address				
**Consult**				

1	1.5 h	A. M. P.—3 h A. P.—2 h E. G.—1.5 h	Brief BCT foundational lecture for patients	Senior Research Manager, National site Research Manager, NL site PE coordinator

Patient responsibilities				
Attend a brief foundational lecture on BCTs. Review a summary of BCTs selected to target the problems identified in the barriers assessment and the process for delivering the intervention. Provide **written feedback** (emailed to Research Manager NL) highlighting your concerns or questions you have with the approach we have selected **within 1–2 weeks** of receiving the summary.				
Researcher commitment				
We will **consider** your feedback and let you know how it influenced the intervention development.				
Senior Research Manager, National site will deliver a short foundational lecture for patients. Research Manager NL will email you a summary of the BCTs selected to target relevant barriers identified in the barriers assessment and the process for delivering the intervention (cc PE coordinator). After we have reviewed your feedback, the PE coordinator will communicate to you how your participation influenced intervention development.				
**Involve**				

2	3 h	A. M. P.—3 h A. P.—5 h A. H.—5 h E. G.—3 h	Brief BCT foundational lecture for patients	Senior Research Manager, national site NL site lead Research Manager, NL PE coordinator

Patient responsibilities				
Review a summary of BCTs selected to target the problems identified in the barriers assessment and the process for delivering the intervention. Provide **written feedback** (emailed to Research Manager) highlighting your concerns or questions you have with the approach we have selected **within 1–2 weeks** of receiving the summary. Attend a round‐table meeting to review your critique and determine how the team can incorporate your feedback.				
Researcher commitment				
We will work with you to **ensure your feedback is incorporated and directly reflected** in the intervention that is developed **to the maximum extent possible**.				
Senior Research Manager, National site will deliver a short foundational lecture for patients. Research Manager, NL will email you draft copies of the materials we have produced (cc PE coordinator). Selected members of the NL team will meet with you to review your feedback and discuss how it can be incorporated.				
**Collaborate**

3–4	4–5 Days (32–40 h) This includes time for training, meetings and independent work	A. M. P.—6 h A. H.—2 days A. P.—2 days N. L. support 2 days E. G.—4 h	Formal BCT training (available online) *Note: This is much more extensive training than the training required for the consult and collaborate levels	Senior Research Manager, National site NL site lead Research manager, NL PE Scientific lead PE coordinator

Patient responsibilities				
Participate in focused conversations, team meetings, and larger stakeholder group meetings to review results of barriers assessment and potential BCTs for this intervention o help to select appropriate BCTs to target the problems identified in the barriers assessment and o plan the process for delivering the intervention. Help to find participants (e.g., patients or others depending on your individual networks) for a larger, key stakeholder group who will be advising on the intervention development and materials moving forward.				
Researcher commitment				
We will work with you to **codevelop** the intervention for the LBP project, relying on your advice and unique perspective. We will **incorporate your advice and recommendations to the maximum extent possible**.				
PE coordinator will advise you on where to find the online BCT training. The Senior Research Manager, national site and the PE coordinator with meet with you to review the training and answer any questions you may have. Subsequent meetings with the NL team and the larger group of key stakeholders will focus on selecting the BCTs and determining how the intervention will be delivered. The Scientific PE Lead will facilitate these meetings.				
**Trial—intervention materials**				
October–December 2020				
Includes developing physician and patient education materials that will be used in intervention delivery (e.g., anything developed for physicians or patients (videos, pamphlets, etc.)				
**Inform**				

0	Patient‐determined	–	None required	PE coordinator

Patient responsibilities				
Review the intervention materials that are selected for the intervention **within 1–2 weeks** of receiving this documentation.				
Researcher commitment				
We will keep you informed by sharing the intervention materials we have selected with you.				
PE coordinator will email you the intervention materials that have been developed/selected for this intervention.				
**Consult**				

0	1.5 h	A. H.—3 h B. F.—2 h A. P.—2 h E. G.—2 h	None required	NL site lead Research Manager, NL PE coordinator Students affiliated with this phase of the project

Patient responsibilities				
Review the intervention materials produced and/or selected for the intervention (may include videos or scripts for family doctors, education or treatment planning materials for patients). Provide **written feedback** (emailed to Research Manager, NL) highlighting the concerns or questions you have about the materials **within 1–2 weeks** of receiving the materials.				
Researcher commitment				
We will **consider** your feedback and let you know how it influenced the intervention development.				
Research Manager, NL will email you the intervention materials (cc PE coordinator). After we have reviewed your feedback the PE coordinator will communicate to you how your participation influenced our intervention materials.				
**Involve**				

1	2.5 h	A. H.—5 h B. F.—3. 5 h A. P.—4 h E. G.—2 h	None required	NL site lead Students affiliated with this phase of the project Research Manager NL PE coordinator

Patient responsibilities				
Review the intervention materials produced and/or selected for the intervention (may include videos or scripts for family doctors, education or treatment planning materials for patients). Provide **written feedback** (emailed to Research Manager NL) highlighting the concerns or questions you have about the materials **within 1–2 weeks** of receiving the materials. Attend a round‐table meeting to review your critique and determine how the team can incorporate your feedback.				
Researcher commitment				
We will work with you to **ensure your feedback is incorporated and directly reflected** in the intervention that is developed **to the maximum extent possible**.				
Research Manager NL will email you draft copies of the materials we have produced (cc PE coordinator) Selected members of the NL team will meet with you to review your feedback and discuss how it can be incorporated.				
**Collaborate**				

2–3	Up to 7 h (includes meeting hours and independent work)	A. H.—2 days A. P.—2 days B. F.—2 days NL SUPPORT—2 days E. G.—4 h	Patient training (TDF)	NL site lead Research Manager NL Students affiliated with this phase of the project Scientific PE Lead PE coordinator

Patient responsibilities				
Attend a lecture on the TDF. Note: This is the same training required at the collaboration level for the barriers assessment. If you have completed the lecture for the barriers assessment, you are not required to repeat it. Partner with researchers and other stakeholders to select and potentially modify or develop intervention materials. This includes: o attending meetings with selected team members to review intervention materials relevant to the BCTs selected during intervention development, o Cocreate new materials and/or modify existing materials (as required), o review and comment on materials selected. Help to find participants (e.g., patients or others depending on your individual networks) for a larger, key stakeholder group who will be advising on the intervention development and materials moving forward.				
Researcher commitment				
We will work with you to **select, modify, and/or create** intervention materials to support the implementation of the selected BCTs, relying on your advice and unique perspective. We will **incorporate your advice and recommendations to the maximum extent possible**.				
The PE coordinator will send you copies of any relevant materials that the team is already aware of for your review before meeting. Subsequent meetings with the NL team and a larger group of key stakeholders will focus on selecting the intervention materials, identifying how they should be modified, and creating new materials as needed. The Scientific PE Lead will facilitate these meetings.				

It is important to note that references to the ‘research team’, acknowledge patients as full partners on the team. The language of ‘researchers’ and ‘patient partners’ is not meant to imply a ‘we’ versus ‘them’ attitude; rather, it is used to clearly delineate the roles and responsibilities of *all* team members. Note also, that while a seven‐step approach is presented, the development of the PEP was not linear. It involved multiple iterations amongst patient partners and researchers, with several drafts specifically informed by patient partners' requests (e.g., information on approximate time commitment or whether training was necessary for a given activity). Throughout the year‐long development of the PEP, the process was presented at multiple national patient partner council meetings and national research team meetings so team members from other provinces could learn from NL efforts. The plan has been well received, and a second site (AB) recently used the template to create its specific provincial PEP. Patient partners have also shared the plan with other research teams with which they work. It was this transferability that led to our decision to publish the process and plan so other research teams could benefit.

### 3.1 A seven‐step approach

#### 3.1.1 Step 1: Choose a framework

At this stage, project researchers and the lead patient partners must decide how they want to engage patients in the research. Using an established patient engagement framework can be an important first step, as this helps shape the approach to patient engagement, clarify its goals and the scope of patients' involvement in the research project. The IAP2 Spectrum of Engagement modified for research^16^ was chosen for this study, as it clearly identified levels of patient involvement (Figure 1) and what the promise as researchers would be to patient partners. It also provided a clear degree of choice to patient partners in how they wished to be involved in specific project activities. This was particularly important, not only because the choice of involvement is the best practice for patient engagement^3^, ^5^, ^8^ but also because patient partners and researchers struggled with the exact nature of engagement for any given project activity. Using the levels of involvement in the research‐modified IAP2 spectrum helped all team members critically reflect on what levels of involvement were possible for each project activity and what the responsibility of each team member would be at any given level.

There are a variety of tools and frameworks in the literature to help plan patient engagement activities in research.^1^, ^2^, ^5^, ^23^ Any of these can be useful guides for research teams, particularly those new to patient engagement as all provide a systematic way of considering the myriad choices to be made in engaging with patient partners in research. However, to our knowledge, there is no consensus in the literature on the ‘best’ tool or framework to use.

The steps that follow remain relevant to any framework that explicitly includes consideration of the levels of patient engagement.^5^, ^23^ For our purposes, we wanted to be as concrete as possible about each and every project activity that could benefit from patient engagement and at what level engagement was possible. The IAP2 spectrum modified for research^16^ allowed us to be explicit about these considerations, and its careful delineation of researcher commitment at each level also prompted us to consider what the parallel commitment for patient partners would be. This specificity proved to be very important to all team members and allowed patient partners to choose at what level they wished to engage in any project activity.

#### 3.1.2 Step 2: Decide which parts of the project need patient engagement

The research team, including all patient partners, needs to review the major project activities and decide where patient input is most needed. For example, project management and execution will include activities, such as participant recruitment, data collection, data analysis and results interpretation. Even within these activities, there are subactivities (e.g., survey development, interview guide development, data entry and analysis and reporting). It is important to list each specific project activity and subactivities.

A useful starting point for identifying project activities is to review the project Gannt chart if one exists. The Gannt chart is an important project management tool and clearly outlines all project activities, when in the project lifecycle they should occur, and how long they should take. The team (including the province's three patient partners) began developing the PEP for the low‐back pain project by reviewing the project activities outlined for the first calendar year of the project and selecting the activities that would benefit most from patient engagement. This level of project activity specificity is very important to patient partners. For example, at the time of starting to create our PEP, there were three main project activities remaining in that year. These included: dissemination of results for the qualitative phase of the study, developing the intervention to be used in the trial and developing the materials to be used in the trial intervention.

Other project activities may also be outlined in the Gannt chart (e.g., project administration) that are not included in our PEP. Regular and ongoing discussion with our patient partners during council meetings revealed their general lack of interest in these activities; as such, they are not included in our final PEP. However, at this stage, patient partners can suggest other activities that come to mind that may not be included in the original project plan. In our project, for example, patient partners acknowledged that they did not want to be involved in weekly operational meetings where site leads discussed new hires, new students or human resource issues. However, in discussion with site leads, they asked to be kept informed of news arising from operational meetings; as such, the team agreed to a weekly email of bulleted points summarizing these discussions.

#### 3.1.3 Step 3: Select potential levels of patient engagement for each project activity requiring patient engagement

Once the team decides on the activities of the project that require/would benefit most from PE, careful thought is needed about at what level engagement can be offered, for *each* project activity. At this stage, select as many levels of engagement as possible to ensure opportunities for higher levels of engagement are not lost. At this point, it is important to address any feasibility considerations. The team must consider time, resources, supports or training required for patients and researchers at each level of the engagement plan, and who will coordinate this study. Higher levels of engagement will necessitate more resources and a greater need for facilitation and general coordination (see Table 1 below for specific examples). This step in the process should be completed in consultation with a patient engagement expert who can advise the team on the nuances of how to operationalize the different levels of engagement. This will allow the team to more fully understand the nature of work and resource needs for each level of engagement, for each project activity. In Canada, we recommend engaging with provincial SUPPORT Units^10^ whose role is to support and build capacity for patient engagement across the country. In the United States and United Kingdom, organisations, such as the Patient Centred Outcomes Research Institute^8^ and INVOLVE,^24^ respectively, are strong advocates of patient and public engagement and offer resources for researchers and patient partners. In discussion with our patient engagement lead (H. E.), we began to critically reflect on what levels were possible for the project activities selected in Step 2. For example, for the knowledge translation activities related to the qualitative phase of the study (known as the ‘barriers assessment’), we acknowledged that the team had sufficient resources to offer all levels of engagement except empowerment (not applicable as patient partners were not solely responsible for carrying out all knowledge translation activities). Ultimately, the team was able to offer all levels except empowerment for each project activity in that calendar year. Future years will require the same type of careful assessment of what resources are available to enact the researcher commitments at each level. We acknowledge that it can be challenging to fully articulate all the levels of engagement possible for *each* project activity, especially if the team does not have access to patient engagement expertise. The templates we provide herein are meant to practically assist teams in this regard. Further, we recommend adequate planning time for this step (and the one to follow). These steps require many conversations with research team members directly involved in each project activity, and with all patient partners as they will have useful feedback during the planning process. For example, during our planning process, patient partners asked whether training would be needed if they wanted to help create intervention materials. This feedback prompted our thinking about the theory behind the project's interventions and a recognition that meaningful contributions to developing interventions could only be possible with an understanding of the theory‐informed intervention development.

#### 3.1.4 Step 4: Describe the levels of patient engagement for researchers and patients

This is perhaps the most important, tangible step in the creation of a PEP. Operationalizing the levels of engagement for each activity is critically important to allow researchers, research staff members and patient partners to fully understand what will be required from all parties to participate at each level of engagement. Ideally, this task will be carried out by a member of the research team who can describe each task or activity that was selected in Step 2, including the nature of the work, training requirements for the patients, facilitation needs of the research team, time commitments, resource needs and the goal of engagement at each of the levels. Budgeting for research support staff with dedicated time to work on planning patient engagement for the project and developing and enacting a PEP is crucial. The descriptions developed at this stage form the bulk of the PEP. For example, the last project activity for that year of the project was the development of intervention materials for the trial (Table 1). It is clear how greater levels of engagement require more time commitments from all team members (Table 1). At the levels of inform and consult, patient partners are asked mainly to review materials and provide written feedback at the consult level. However, at the collaborate level, patient partners are asked to help design materials, attend meetings where intervention materials will be reviewed, and attend a training session specific to the theoretical framework that guides intervention development. The researcher commitments and time required are also greater at this level.

As for Step 3, it can be challenging to operationalize the levels of engagement in a concrete way: research is unpredictable, timelines change and experienced researchers may not consciously think about the minutiae of tasks associated with various research activities. Ongoing conversations and brainstorming with relevant researchers and all patient partners are necessary to be as concrete as possible. It is the drafting of plans following such conversations and reviews by team members that highlight when something is unclear and generate questions. These questions ultimately help ensure that relevant information is included in the operationalization of the various levels.

#### 3.1.5 Step 5: Review with project researchers and research support staff

Once the engagement levels for each activity have been fully operationalized, review those descriptions with researchers and research staff to discuss in detail and decide if the plan is feasible. Named team members should consider whether they can accommodate the researcher commitments outlined for each level of engagement and if the team has the resources to commit to all levels of engagement. At this stage, revisions to previous choices might be needed to narrow down the levels of engagement initially planned. For example, researchers may find more value in offering higher levels of engagement to patients for activities such as developing patient‐facing materials than they would for monitoring day‐to‐day project operations.

#### 3.1.6 Step 6: Review with patient partners

Patient partners also need to review the selected project activities and the levels of engagement that will be offered. If your research team does not yet have patient partners, we recommend reaching out to local patient engagement resources (such as the SUPPORT Units in Canada) or at the least, local public interest, community or advisory groups. At this stage, public feedback is needed about the clarity of activity descriptions and levels of engagement to ensure that the patient and researcher roles and responsibilities are easily understood and delineated. This really is critical. In our project, we were fortunate to have three lead patient partners from the very beginning of the project who could advise on all these steps thus far. As new partners joined the patient partner council, they also reviewed the plan. Their feedback on the specific project activities and levels of engagement for each resulted in numerous modifications to original descriptions. For example, patient partner review resulted in adding clarifying information about time commitment estimates, whether training would be necessary for any given project activities and a time estimate for it, and exactly which research team member would be responsible for following up with patient partners (and by when). These granular details matter.

#### 3.1.7 Step 7: Survey your patient partners for their choices

Once the entire research team, including partner partners, have reviewed and agreed on the PEP, patient partners are asked to choose how they want to be involved in the project activities outlined. The template itself functioned as a survey to allow patient partners an organized and formal method of identifying the project activities they were most interested in and at what level they wished to engage with those activities. The final version of the template (Table 1) was provided to each provincial patient partner, who recorded their preferences for each project activity. This allows both patients and researchers to retain a record of preferences for each patient partner that can be reviewed at any time throughout the project or revisited in the event of project changes. To this end, we recommend reviewing the PEP at least annually and as necessary in the event of unplanned changes to the project.

In our project, we were fortunate to have adequate levels of patient partner interest across all project activities and most levels of involvement. However, if we take seriously the notion of offering choice to patient partners and flexibility in how they wish to be involved (and at what level), then it is possible some project activities or levels of involvement may receive little or no interest from patient partners. If this is the case, but the team strongly believes a certain level of involvement is necessary for any given project activity, additional recruitment of patient partners might be needed. Again, we recommend reaching out to local patient engagement resources (such as the SUPPORT Units in Canada) or at the least, local public interest, community, patient or advisory groups with a clear description of the patient partner opportunity, including a description of the level of involvement desired. In the event that a desired level of involvement was not achieved for project activity, it is important to be transparent about this in any knowledge translation outputs and reflect on what the implications are.

### 3.2 A template for others to follow

Below, a fillable PEP template is presented (Figure 2). It highlights the kind of information that should be crafted for each section of the plan, regardless of the research project. It provides a guide for others to follow and should encourage critical and deep reflection on specific project activities, possible levels of engagement for each activity and resources available to enact engagement.

#### 3.2.1 Evaluating the plan

The evaluation of patient engagement remains understudied, making it difficult to know what works best and when.^5^, ^13^, ^14^ The research team will evaluate the use of our PEP for year one before finalizing plans for subsequent project years. While the COVID‐19 pandemic has slowed or halted data collection in all three study sites, an unforeseen benefit has been the luxury of time to create a detailed PEP and its evaluation. The team anticipates a mixed‐method participatory evaluation, which some team members have already used successfully in evaluating patient‐oriented research.^14^ We anticipate data collection through the use of online surveys and interviews, as well as in the collection of administrative, descriptive data (e.g., were follow up emails sent to patient partners as outlined in the plan). Key evaluation questions will potentially include:

Did all team members meet all commitments as outlined in the plan?

Was any training provided sufficient to enable team members to engage at the levels they preferred?

Were there any setbacks/lessons learned? What could be improved about patient engagement activities?

What was the patient and researcher experience of enacting the plan? What could we do differently?

Did other project sites use our template? Did patient partners share the plan with other project teams with whom they work?

### 3.3 Conclusions and lessons learned

Engaging with patient partners in health research is now largely accepted, and growing evidence supports its ability to improve research relevance, quality and outcomes.^1^, ^11^, ^12^, ^14^ Despite a growing number of tools and frameworks, however, there remains relatively little tangible guidance for how to operationalize the levels of possible engagement across health research activities. The detailed PEP provided here is a practical template that can be used widely by other health researchers and patient partners, customized to fit any specific project. We conclude with key lessons learned in the process of creating the plan.

First, *Anticipate and budget for costs*—The successful creation and implementation of a PEP requires targeted resources. In particular, a dedicated research staff person who coordinates the process is desired; budget for such research support at the funding application stage. Second, *Be transparent*—It is critical to identify those areas where patient engagement will not be practically possible in a project before creating the plan (e.g., members of our patient partner council who were recruited after funding was obtained had no opportunity to advise on study research questions or the theoretical framework that guides the study as these decisions were already made). Also identify if a particular *level* of engagement is not possible for all specific project activities (e.g., empowerment was not available for the project activities of staff hiring or financial management and reporting, nor for most research activities). Third, recognize that *Specifics matter*—Patient preferences can change when they are given additional information. For example, all three of our patient partners had initially chosen the collaborate level of involvement for many project activities in earlier verbal discussions and in earlier (less detailed) versions of the PEP. Through iterative drafts and reviews of the template, however, and at patient partners' request, we added additional information about training and time commitments. When this more precise information was included, our patient partners chose the consult or involve level of engagement instead. We believe this level of detail is important if we are to foster the levels of engagement patients truly wish. *Cocreate*—While we highly endorse cocreating PEPs with patient partners, we recommend researchers and research staff create the initial draft. These are the team members who best know specific project details and timelines, both of which are crucial to formulating a PEP. Once that initial draft is shared, however, patient partners are the team members who will then best be able to identify missing information gaps, and their feedback must drive all further iterations. Finally, *Be flexible*—If the COVID‐19 pandemic has taught us anything, it is to be flexible. In this project, the pandemic did alter data collection activities, particularly in the Ontario and Alberta sites. It is important to discuss with patient partners whether alternative activities are available or update timelines regularly. Our council has continued to meet regularly throughout the pandemic, which has been important to maintain trust, communication and motivation. It also provides the opportunity for social support and brainstorming new ideas (e.g., creating potential knowledge translation outputs related to patient engagement, while waiting for data collection activities to resume in full).

In this paper, we aimed to provide guidance on how to codesign and operationalize a detailed PEP for any health research project. We not only include a fully worked example of a PEP using our trial as an example but also provide a fillable template for others to use in any health research project. We hope this guide can serve as a resource for research funders, researchers, providers and patient partners with the goal of advancing a more rigorous approach to the development and implementation of patient engagement in health research.

## CONFLICT OF INTERESTS

The authors declare that there are no conflict of interests.

## AUTHOR CONTRIBUTIONS

Etchegary, Pike and Hall conceived the planning template, with the approval of patient partners, and created an initial draft. Patient partner coauthors, Johnston, Francis and Goold reviewed the initial draft and contributed to revisions. All authors reviewed the patient‐approved initial draft and contributed important intellectual content over multiple iterations. All authors reviewed and approved the final planning template presented here. Etchegary drafted the manuscript; all authors reviewed multiple drafts and contributed intellectual content to revisions and all approved the final version.

## Data Availability

Data sharing is not applicable to this article as no datasets were generated or analysed during the current study.
